# A New Species of *Fleischmannia* (Asteraceae, Eupatorieae) from El Salvador

**DOI:** 10.3897/phytokeys.7.2088

**Published:** 2011-11-29

**Authors:** Harold Robinson

**Affiliations:** 1 Dept. of Botany, MRC 166, National Museum of Natural History, P.O. Box 37012, Smithsonian Institution, Washington D.C. 20013-7012

**Keywords:** *Fleischmannia*, Eupatorieae, Asteraceae, Mesoamerica, El Salvador

## Abstract

*Fleischmannia profusa* is named as new from El Salvador based on material with deltoid leaf blades, numerous axillary fascicles of leaves on the vegetative stems and c. 60 sharply obviously acuminate involucral bracts in 4-5 strongly gradate series.

## Introduction

Since the redefinition of the limits of the genus *Fleischmannia* Sch.Bip. (King and Robinson 1966, 1970) numerous studies have added species to the genus for the Flora Mesoamerica area (King and Robinson 1972, 1974, 1975, 1978, 1991; Robinson 2001). A further revision of the manuscript for the Eupatorieae of Mesoamerica has revealed an additional distinctive species of *Fleischmannia* in need of description. The species is named “profusa” because of the numerous small axillary fascicles of leaves on the vegetative stems and because of the numerous sharply pointed involucral bracts in many gradate series. This new species is described below.

## Taxonomy

### Fleischmannia profusa

H. Rob., sp. nov.

urn:lsid:ipni.org:names:77115898-1

http://species-id.net/wiki/Fleischmannia_profusa

Figure 1 

#### Type.

El Salvador. Prov. La Libertad, Fls. purple, herb 0.5 m, common on rocky slopes along litoral road to La Libertad, alt. 30 m, s.d. *A. Molina, W.C. Burger & B. Wallenta 16685* (holotype US, isotype F).

Ab species *Fleischmanniam* aliam omninoin phyllariis numerosis 4-5 seriatis argute acuminatis distincte gradatis differt.

Branching herbs to 0.5 m; stems hispidulous with minute erect stipitate glands, glabrescent below; internodes mostly 1.5-2.0 cm. Leaves opposite, with numerous fascicles in axils; petiole 3-6 mm; blade mostly 2.0-3.2 × 1.4-2.3 cm, deltoid, trinervate from base, surface with few to many minute stipitate glands, without glandular dots, adaxial surface sparsely pilose, base broadly subtruncate, margins 5-8-crenate beyond widest part, apex short-acute. Capitulescence of 1-5 capitula terminal on main stem and branches, subtended by sparse narrow bracteoles 3-7 mm; peduncles 0.6-1.0 mm, with minute stipitate glands. Capitula 6-7 mm; phyllaries c. 60, subimbricate, graduate in c. 4-5 series, lanceolate, 1.5-4.0 × 0.4-08 mm, all narrowly acute to slightly acuminate, green, scarcely scarious, with many minute stipitate glands. Florets c. 60; corollas c. 3 mm, purple, lobes c. 0.4 mm, with few or no small trichomes; style branches not broadened distally. Cypselae c. 1.5 mm, black with black ribs at maturity, scabrid on ribs; pappus with c. 20 bristles 2.5-3.8 mm, slightly non-contiguous at base. *Common on rocky slopes along litoral road, 30 m.* ES (*Molina, Burger & Wallenta 16685* (US).

The type specimen was originally distributed from the Escuela Agricola Panamericana and the Chicago Natural History museum under the name *Eupatorium ovillum* Standl. & Steyerm, a completely different species now known as *Ageratina ovilla* (Standl. & Steyerm.) R.M. King & H. Rob. In the initial study of the Mesoamerican Eupatorieae, the specimen described here as a new species was included in the widely distributed *Fleischmannia imitans* (B.L. Rob.) R.M. King & H. Rob. with which it shares the achenes with blackened ribs, the pubescence of numerous stipitate glands and the numerous pointed involucral bracts. The bracts of the latter, however, are only 35-40 in ca. 3 weakly subimbricate series, are not as obviously acuminate and are not strongly unequal or gradate. The leaves of the new species are primarily deltoid while those of *Fleischmannia imitans* are ovate lanceolate to lanceolate. The latter also has no or comparatively few axillary fascicles of leaves on the vegetative stems.

**Figure 1.**
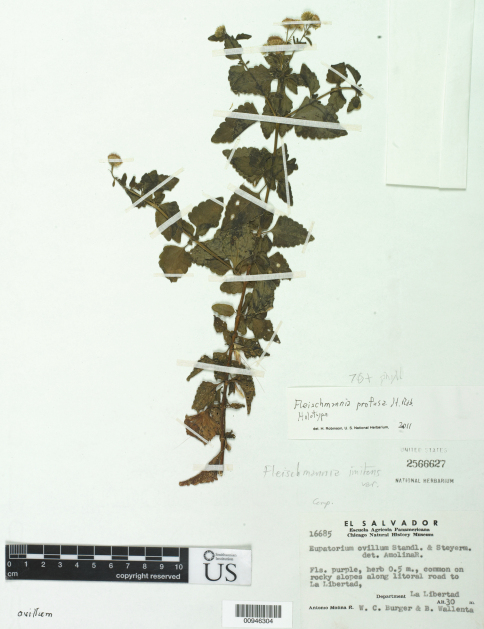
*Fleischmannia profusa* H. Rob., holotype (US).

The distinctions between *Fleischmannia profusa* and *Fleischmannia imitans* in couplet form are as follows:

**Table:** 

1	Leaves ovate-lanceolate to lanceolate; phyllaries 35-40 in c. 3 weakly subimbricate series, appearing superficially eximbricate; stems with few or no axillary fascicles of leaflets	*Fleischmannia imitans*
–	Leaves deltoid; phyllaries ca. 60 in c. 4-5- gradate distinctly subimbricate series; stems with many axillary fascicles of leaflets	*Fleischmannia profusa*

## Supplementary Material

XML Treatment for Fleischmannia profusa
